# 5,7-Dibromo-3-trifluoro­methyl-3,4-dihydro­acridin-1(2*H*)-one

**DOI:** 10.1107/S1600536811027516

**Published:** 2011-07-16

**Authors:** Cosmas O. Okoro, Tasneem Siddiquee, Olugbeminiyi O. Fadeyi

**Affiliations:** a Department of Chemistry, Boswell Science Complex, Tennessee State University, Nashville, 3500 John A Merritt Blvd, Nashville, TN 37209, USA

## Abstract

In the title compound, C_14_H_8_Br_2_F_3_NO, the mol­ecule is disordered across an approximate non-crystallographic mirror plane, which is in the plane of the fused ring system [The tetrahedral C atom bearing the trifluormethyl substituent is disordered with site occupancy factors of 0.80 (2) and 0.20 (2)]. In the crystal, a one-dimensional stacking of mol­ecules involves inter­actions between the pyridine ring and symmetry-related Br and O atoms of adjacent mol­ecules. The stacking distance between the mean planes of adjacent mol­ecules is 3.395 (4) Å.

## Related literature

For the anti­cancer activity of the title compound, see: Fadeyi *et al.* (2008 ▶). For fluorinated acridones, see: Fadeyi *et al.* (2008 ▶); Mayur *et al.* (2009 ▶); Svyatkina *et al.* (1988 ▶). For a related structure, see: Martinez *et al.* (1995 ▶).
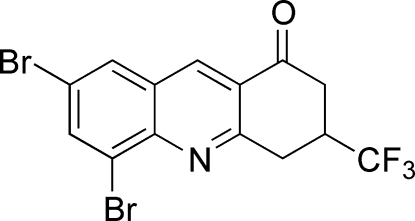


## Experimental

### 

#### Crystal data

C_14_H_8_Br_2_F_3_NO
*M* *_r_* = 423.03
Triclinic, 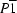

*a* = 5.3303 (10) Å
*b* = 10.926 (2) Å
*c* = 12.354 (2) Å
α = 83.349 (6)°
β = 85.741 (6)°
γ = 85.051 (6)°
*V* = 710.5 (2) Å^3^
*Z* = 2
Mo *K*α radiation
μ = 5.74 mm^−1^
*T* = 293 K
0.21 × 0.13 × 0.06 mm

#### Data collection

Rigaku XtaLAB mini diffractometer
Absorption correction: multi-scan (*CrystalClear*; Rigaku, 1999 ▶; Pflugrath, 1999 ▶) *T*_min_ = 0.379, *T*_max_ = 0.725
4444 measured reflections
3153 independent reflections
2188 reflections with *I* > 2σ(*I*)
*R*_int_ = 0.050

#### Refinement

*R*[*F* ^2^ > 2σ(*F* ^2^)] = 0.055
*wR*(*F* ^2^) = 0.134
*S* = 0.97
3153 reflections
217 parameters
34 restraints
H-atom parameters constrained
Δρ_max_ = 0.80 e Å^−3^
Δρ_min_ = −0.67 e Å^−3^

### 

Data collection: *CrystalClear* (Rigaku, 1999 ▶; Pflugrath, 1999 ▶); cell refinement: *CrystalClear*; data reduction: *CrystalClear*; program(s) used to solve structure: *SIR2004* (Burla *et al.*, 2005 ▶); program(s) used to refine structure: *SHELXL97* (Sheldrick, 2008 ▶); molecular graphics: *ORTEP-3 for Windows* (Farrugia, 1997 ▶) as included in *WinGX* (Farrugia, 1999 ▶); software used to prepare material for publication: *WinGX* and *publCIF* (Westrip, 2010 ▶).

## Supplementary Material

Crystal structure: contains datablock(s) I, global. DOI: 10.1107/S1600536811027516/pk2334sup1.cif

Structure factors: contains datablock(s) I. DOI: 10.1107/S1600536811027516/pk2334Isup2.hkl

Supplementary material file. DOI: 10.1107/S1600536811027516/pk2334Isup3.cml

Additional supplementary materials:  crystallographic information; 3D view; checkCIF report

## Figures and Tables

**Fig. 1.**
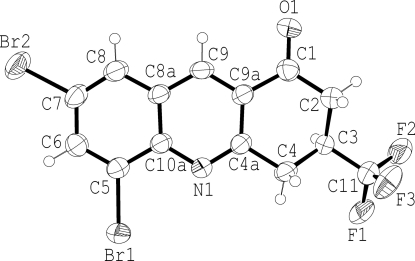
Thermal ellipsoid plot (50% probability) of 5,7-dibromo-3-(trifluoromethyl)-3,4-dihydroacridin-1(2H)-one. In the interest of clarity, only the major component of disorder is shown.

## supplementary crystallographic information

### Comment

The compound, 5,7-dibromo-3-trifluoromethyl-3,4-dihydroacridin-1(2*H*)-one exhibited anticancer activity in several cell lines (Fadeyi *et al.*, 2008).  The molecule is disordered across an approximate  non-crystallographic mirror plane, which is in the plane of the fused ring system.  A one dimensional stacking of molecules involves interactions between the pyridine ring and symmetry related Br (*via* 1+x, y, z) and O (*via* (1-x, y, z) on adjacent molecules.  The stacking distance between the mean planes of adjacent molecules is 3.395 (4) Å.  The molecule is disordered in the crystal with  site occupancy factors of 0.796 (6) and 0.204 (6) for the major and minor components, respectively.

### Experimental

To a mixture of 3,5-dibromo-2-aminobenzaldehyde (1.0 mmol) and 5-trifluoromethyl-cyclohexanedione (1.0 mmol) was added 1 mL of 1N HCl. The reaction mixture was stirred at 60–75°C for 30 minutes. After this period the reaction mixture was neutralized with 1 mL of 1N NaOH.  The solid was filtered and washed with water (3 × 6 mL) and air dried. Colorless single crystals suitable for X-ray diffraction studies were harvested after recrystallization from aqueous ethanol. M.p. = 140–143°C. IR (nujol): 2925, 1600, 1465, 964 and 787 cm^-1^. ^1^HNMR (CDCl_3_): δ 2.4–2.49 (dd, 2H), 2.88–2.93 (dd, 2H), 3.22 (m, 1H), 8.32 (d, 1H), 8.91 (s, 1H).

### Refinement

H atoms (except those of the minor disorder component) were found in difference Fourier maps and subsequently placed in idealized positions with constrained distances of 0.97 Å (*R*_2_CH_2_), 0.98 Å (*R*_3_CH), 0.93 Å (C_sp2_H), and with *U*_iso_(H) values set to 1.2*U*_eq_ of the attached atom.

To ensure satisfactory refinement of the disordered parts of the structure, a combination of constraints and restraints were needed. The constraints (*SHELXL97* command EADP) were used to make the displacement parameters of closely proximate disordered atoms equal.  The restraints (*SHELXL97* commands SAME, SIMU & DELU) were used to ensure similar geometries and displacement parameters of closely proximate, chemically identical groups.

### Crystal data

**Table:**

C_14_H_8_Br_2_F_3_NO	*Z* = 2
*M**_r_* = 423.03	*F*(000) = 408
Triclinic, *P*1	*D*_x_ = 1.977 Mg m^−^^3^
Hall symbol: -P 1	Mo *K*α radiation, λ = 0.71073 Å
*a* = 5.3303 (10) Å	Cell parameters from 3749 reflections
*b* = 10.926 (2) Å	θ = 3.3–27.6°
*c* = 12.354 (2) Å	µ = 5.74 mm^−^^1^
α = 83.349 (6)°	*T* = 293 K
β = 85.741 (6)°	Prism, colorless
γ = 85.051 (6)°	0.21 × 0.13 × 0.06 mm
*V* = 710.5 (2) Å^3^	

### Data collection

**Table:**

Rigaku XtaLAB mini diffractometer	2188 reflections with *I* > 2σ(*I*)
graphite	*R*_int_ = 0.050
ω scans	θ_max_ = 27.5°, θ_min_ = 3.3°
Absorption correction: multi-scan (*CrystalClear*; Rigaku, 1999; Pflugrath, 1999)	*h* = −3→6
*T*_min_ = 0.379, *T*_max_ = 0.725	*k* = −14→14
4444 measured reflections	*l* = −15→15
3153 independent reflections	

### Refinement

**Table:**

Refinement on *F*^2^	Primary atom site location: structure-invariant direct methods
Least-squares matrix: full	Secondary atom site location: difference Fourier map
*R*[*F*^2^ > 2σ(*F*^2^)] = 0.055	Hydrogen site location: inferred from neighbouring sites
*wR*(*F*^2^) = 0.134	H-atom parameters constrained
*S* = 0.97	*w* = 1/[σ^2^(*F*_o_^2^) + (0.0643*P*)^2^] where *P* = (*F*_o_^2^ + 2*F*_c_^2^)/3
3153 reflections	(Δ/σ)_max_ = 0.001
217 parameters	Δρ_max_ = 0.80 e Å^−^^3^
34 restraints	Δρ_min_ = −0.67 e Å^−^^3^

### Special details

**Table:**

Geometry. All s.u.'s (except the s.u. in the dihedral angle between two l.s. planes) are estimated using the full covariance matrix. The cell s.u.'s are taken into account individually in the estimation of s.u.'s in distances, angles and torsion angles; correlations between s.u.'s in cell parameters are only used when they are defined by crystal symmetry. An approximate (isotropic) treatment of cell s.u.'s is used for estimating s.u.'s involving l.s. planes.
Refinement. Refinement of *F*^2^ against ALL reflections. The weighted *R*-factor *wR* and goodness of fit *S* are based on *F*^2^, conventional *R*-factors *R* are based on *F*, with *F* set to zero for negative *F*^2^. The threshold expression of *F*^2^ > 2σ(*F*^2^) is used only for calculating *R*-factors(gt) *etc*. and is not relevant to the choice of reflections for refinement. *R*-factors based on *F*^2^ are statistically about twice as large as those based on *F*, and *R*- factors based on ALL data will be even larger.

### Fractional atomic coordinates and isotropic or equivalent isotropic displacement parameters (Å^2^)

**Table:**

	*x*	*y*	*z*	*U*_iso_*/*U*_eq_	Occ. (<1)
Br1	−0.08381 (9)	0.38272 (5)	0.91649 (4)	0.05150 (19)	
Br2	0.25701 (14)	0.07810 (5)	0.58890 (5)	0.0731 (2)	
N1	0.3349 (7)	0.5389 (3)	0.8099 (3)	0.0420 (9)	
O1	1.0453 (7)	0.6599 (4)	0.5769 (3)	0.0583 (10)	
C1	0.8841 (9)	0.6822 (5)	0.6495 (4)	0.0455 (11)	
C2	0.875 (2)	0.7995 (11)	0.7024 (11)	0.051 (3)	0.796 (6)
H2A	0.7635	0.8621	0.6642	0.061*	0.796 (6)
H2B	1.0423	0.8292	0.6969	0.061*	0.796 (6)
C3	0.7812 (11)	0.7788 (5)	0.8223 (5)	0.0418 (14)	0.796 (6)
H3	0.8977	0.7168	0.8602	0.05*	0.796 (6)
C11	0.770 (2)	0.8972 (8)	0.8778 (9)	0.056 (2)	0.796 (6)
F1	0.6920 (9)	0.8785 (4)	0.9826 (3)	0.0747 (14)	0.796 (6)
F2	1.0046 (8)	0.9352 (4)	0.8774 (4)	0.0779 (15)	0.796 (6)
F3	0.6313 (10)	0.9893 (4)	0.8297 (4)	0.0829 (18)	0.796 (6)
C4	0.519 (3)	0.7294 (10)	0.8318 (9)	0.048 (3)	0.796 (6)
H4A	0.4667	0.7103	0.9084	0.058*	0.796 (6)
H4B	0.3987	0.7933	0.8007	0.058*	0.796 (6)
C2'	0.897 (9)	0.779 (5)	0.718 (5)	0.051 (3)	0.204 (6)
H2'1	0.9643	0.8496	0.6739	0.061*	0.204 (6)
H2'2	1.0123	0.7504	0.7741	0.061*	0.204 (6)
C3'	0.641 (4)	0.8190 (18)	0.7713 (17)	0.044 (5)*	0.204 (6)
H3'	0.5204	0.8398	0.7148	0.053*	0.204 (6)
C11'	0.661 (4)	0.933 (2)	0.831 (2)	0.058 (7)*	0.204 (6)
F1'	0.442 (3)	0.9642 (17)	0.8836 (14)	0.084 (6)*	0.204 (6)
F2'	0.828 (6)	0.897 (3)	0.910 (2)	0.095 (12)*	0.204 (6)
F3'	0.754 (4)	1.0247 (17)	0.7699 (17)	0.093 (7)*	0.204 (6)
C4'	0.546 (13)	0.714 (4)	0.853 (5)	0.048 (3)	0.204 (6)
H4'1	0.3875	0.7386	0.8911	0.058*	0.204 (6)
H4'2	0.6692	0.6838	0.9059	0.058*	0.204 (6)
C4A	0.5120 (8)	0.6143 (4)	0.7741 (4)	0.0421 (11)	
C5	0.1374 (9)	0.3514 (4)	0.7950 (4)	0.0426 (10)	
C6	0.1224 (9)	0.2471 (4)	0.7460 (4)	0.0489 (12)	
H6	0.0023	0.1921	0.7718	0.059*	
C7	0.2894 (10)	0.2233 (4)	0.6562 (4)	0.0506 (12)	
C8	0.4680 (10)	0.3008 (4)	0.6163 (4)	0.0498 (12)	
H8	0.5753	0.2832	0.5563	0.06*	
C8A	0.4892 (9)	0.4080 (4)	0.6668 (3)	0.0410 (10)	
C9	0.6745 (9)	0.4917 (4)	0.6312 (4)	0.0436 (11)	
H9	0.7869	0.4772	0.5719	0.052*	
C9A	0.6878 (9)	0.5944 (4)	0.6845 (3)	0.0410 (10)	
C10A	0.3249 (8)	0.4357 (4)	0.7576 (4)	0.0408 (10)	

### Atomic displacement parameters (Å^2^)

**Table:**

	*U*^11^	*U*^22^	*U*^33^	*U*^12^	*U*^13^	*U*^23^
Br1	0.0544 (3)	0.0519 (3)	0.0473 (3)	−0.0081 (2)	0.0128 (2)	−0.0092 (2)
Br2	0.1173 (6)	0.0430 (3)	0.0626 (4)	−0.0170 (3)	0.0078 (3)	−0.0210 (3)
N1	0.042 (2)	0.043 (2)	0.042 (2)	−0.0072 (18)	0.0037 (17)	−0.0121 (17)
O1	0.058 (2)	0.060 (2)	0.056 (2)	−0.0144 (18)	0.0230 (18)	−0.0146 (18)
C1	0.043 (2)	0.044 (3)	0.048 (3)	−0.002 (2)	0.002 (2)	−0.005 (2)
C2	0.053 (4)	0.047 (5)	0.053 (5)	−0.008 (3)	0.011 (4)	−0.011 (4)
C3	0.048 (3)	0.034 (3)	0.044 (3)	−0.009 (3)	0.007 (3)	−0.010 (2)
C11	0.057 (5)	0.044 (4)	0.069 (5)	−0.007 (3)	0.007 (5)	−0.018 (4)
F1	0.104 (3)	0.064 (3)	0.061 (2)	−0.014 (2)	0.013 (2)	−0.035 (2)
F2	0.070 (3)	0.073 (3)	0.099 (3)	−0.021 (2)	0.009 (2)	−0.046 (3)
F3	0.107 (4)	0.046 (3)	0.097 (4)	0.017 (3)	−0.016 (3)	−0.026 (3)
C4	0.042 (4)	0.060 (4)	0.049 (6)	−0.009 (4)	0.005 (4)	−0.026 (4)
C2'	0.053 (4)	0.047 (5)	0.053 (5)	−0.008 (3)	0.011 (4)	−0.011 (4)
C4'	0.042 (4)	0.060 (4)	0.049 (6)	−0.009 (4)	0.005 (4)	−0.026 (4)
C4A	0.042 (2)	0.042 (3)	0.043 (2)	−0.003 (2)	−0.001 (2)	−0.013 (2)
C5	0.046 (2)	0.045 (3)	0.037 (2)	−0.002 (2)	−0.0009 (19)	−0.008 (2)
C6	0.058 (3)	0.042 (3)	0.046 (3)	−0.009 (2)	0.002 (2)	−0.001 (2)
C7	0.072 (3)	0.034 (3)	0.047 (3)	−0.002 (2)	0.000 (2)	−0.011 (2)
C8	0.061 (3)	0.046 (3)	0.042 (3)	−0.001 (2)	0.006 (2)	−0.011 (2)
C8A	0.050 (3)	0.042 (3)	0.032 (2)	−0.003 (2)	0.0028 (19)	−0.0085 (19)
C9	0.048 (3)	0.044 (3)	0.036 (2)	0.002 (2)	0.011 (2)	−0.007 (2)
C9A	0.045 (2)	0.040 (3)	0.038 (2)	−0.002 (2)	0.001 (2)	−0.009 (2)
C10A	0.045 (3)	0.038 (2)	0.038 (2)	0.000 (2)	0.001 (2)	−0.0072 (19)

### Geometric parameters (Å, °)

**Table:**

Br1—C5	1.884 (4)	C2'—H2'2	0.97
Br2—C7	1.901 (5)	C3'—C4'	1.54 (2)
N1—C4A	1.325 (6)	C3'—C11'	1.538 (19)
N1—C10A	1.369 (5)	C3'—H3'	0.98
O1—C1	1.226 (5)	C11'—F3'	1.29 (2)
C1—C2'	1.43 (5)	C11'—F1'	1.33 (2)
C1—C9A	1.487 (7)	C11'—F2'	1.37 (2)
C1—C2	1.499 (13)	C4'—C4A	1.57 (6)
C2—C3	1.526 (11)	C4'—H4'1	0.97
C2—H2A	0.97	C4'—H4'2	0.97
C2—H2B	0.97	C4A—C9A	1.420 (6)
C3—C11	1.527 (9)	C5—C6	1.361 (6)
C3—C4	1.535 (13)	C5—C10A	1.433 (6)
C3—H3	0.98	C6—C7	1.403 (6)
C11—F3	1.307 (12)	C6—H6	0.93
C11—F1	1.327 (11)	C7—C8	1.356 (7)
C11—F2	1.353 (11)	C8—C8A	1.406 (6)
C4—C4A	1.519 (13)	C8—H8	0.93
C4—H4A	0.97	C8A—C9	1.415 (7)
C4—H4B	0.97	C8A—C10A	1.416 (6)
C2'—C3'	1.52 (2)	C9—C9A	1.375 (6)
C2'—H2'1	0.97	C9—H9	0.93
			
C4A—N1—C10A	117.5 (4)	F3'—C11'—F1'	113 (2)
O1—C1—C2'	123.2 (16)	F3'—C11'—F2'	106 (2)
O1—C1—C9A	120.2 (5)	F1'—C11'—F2'	106 (2)
C2'—C1—C9A	115.7 (16)	F3'—C11'—C3'	113.6 (19)
O1—C1—C2	121.1 (5)	F1'—C11'—C3'	110.7 (17)
C9A—C1—C2	118.6 (5)	F2'—C11'—C3'	106 (2)
C1—C2—C3	111.0 (8)	C3'—C4'—C4A	101 (3)
C1—C2—H2A	109.4	C3'—C4'—H4'1	111.6
C3—C2—H2A	109.4	C4A—C4'—H4'1	111.6
C1—C2—H2B	109.4	C3'—C4'—H4'2	111.6
C3—C2—H2B	109.4	C4A—C4'—H4'2	111.6
H2A—C2—H2B	108	H4'1—C4'—H4'2	109.4
C2—C3—C11	112.0 (7)	N1—C4A—C9A	123.6 (4)
C2—C3—C4	109.8 (8)	N1—C4A—C4	116.9 (5)
C11—C3—C4	109.5 (6)	C9A—C4A—C4	119.5 (5)
C2—C3—H3	108.5	N1—C4A—C4'	113.6 (13)
C11—C3—H3	108.5	C9A—C4A—C4'	121.7 (16)
C4—C3—H3	108.5	C6—C5—C10A	120.9 (4)
F3—C11—F1	109.6 (7)	C6—C5—Br1	119.7 (4)
F3—C11—F2	106.5 (7)	C10A—C5—Br1	119.4 (3)
F1—C11—F2	104.9 (10)	C5—C6—C7	119.6 (5)
F3—C11—C3	113.9 (9)	C5—C6—H6	120.2
F1—C11—C3	111.9 (6)	C7—C6—H6	120.2
F2—C11—C3	109.5 (6)	C8—C7—C6	122.1 (4)
C4A—C4—C3	112.7 (8)	C8—C7—Br2	119.9 (4)
C4A—C4—H4A	109.1	C6—C7—Br2	118.0 (4)
C3—C4—H4A	109.1	C7—C8—C8A	119.2 (4)
C4A—C4—H4B	109.1	C7—C8—H8	120.4
C3—C4—H4B	109.1	C8A—C8—H8	120.4
H4A—C4—H4B	107.8	C8—C8A—C9	122.4 (4)
C1—C2'—C3'	113 (4)	C8—C8A—C10A	120.6 (4)
C1—C2'—H2'1	109	C9—C8A—C10A	117.0 (4)
C3'—C2'—H2'1	109	C9A—C9—C8A	119.8 (4)
C1—C2'—H2'2	109	C9A—C9—H9	120.1
C3'—C2'—H2'2	109	C8A—C9—H9	120.1
H2'1—C2'—H2'2	107.8	C9—C9A—C4A	118.7 (4)
C2'—C3'—C4'	110 (2)	C9—C9A—C1	120.6 (4)
C2'—C3'—C11'	110 (2)	C4A—C9A—C1	120.7 (4)
C4'—C3'—C11'	109 (2)	N1—C10A—C8A	123.4 (4)
C2'—C3'—H3'	108.9	N1—C10A—C5	119.0 (4)
C4'—C3'—H3'	108.9	C8A—C10A—C5	117.6 (4)
C11'—C3'—H3'	108.9		
			
O1—C1—C2—C3	−147.1 (7)	C10A—C5—C6—C7	−1.3 (7)
C2'—C1—C2—C3	−43 (9)	Br1—C5—C6—C7	−179.3 (4)
C9A—C1—C2—C3	34.9 (12)	C5—C6—C7—C8	0.3 (8)
C1—C2—C3—C11	−179.8 (8)	C5—C6—C7—Br2	−178.9 (4)
C1—C2—C3—C4	−58.0 (11)	C6—C7—C8—C8A	0.6 (8)
C2—C3—C11—F3	56.1 (10)	Br2—C7—C8—C8A	179.8 (4)
C4—C3—C11—F3	−65.8 (9)	C7—C8—C8A—C9	178.6 (5)
C2—C3—C11—F1	−178.9 (10)	C7—C8—C8A—C10A	−0.6 (7)
C4—C3—C11—F1	59.1 (11)	C8—C8A—C9—C9A	−179.3 (4)
C2—C3—C11—F2	−63.0 (11)	C10A—C8A—C9—C9A	−0.2 (7)
C4—C3—C11—F2	175.0 (8)	C8A—C9—C9A—C4A	−0.5 (7)
C2—C3—C4—C4A	54.2 (10)	C8A—C9—C9A—C1	178.9 (4)
C11—C3—C4—C4A	177.5 (7)	N1—C4A—C9A—C9	0.2 (7)
O1—C1—C2'—C3'	159 (2)	C4—C4A—C9A—C9	−178.2 (7)
C9A—C1—C2'—C3'	−32 (5)	C4'—C4A—C9A—C9	168 (3)
C2—C1—C2'—C3'	76 (10)	N1—C4A—C9A—C1	−179.1 (4)
C1—C2'—C3'—C4'	66 (5)	C4—C4A—C9A—C1	2.5 (9)
C1—C2'—C3'—C11'	−173 (3)	C4'—C4A—C9A—C1	−12 (3)
C2'—C3'—C11'—F3'	55 (4)	O1—C1—C9A—C9	−4.2 (7)
C4'—C3'—C11'—F3'	176 (3)	C2'—C1—C9A—C9	−174 (3)
C2'—C3'—C11'—F1'	−176 (3)	C2—C1—C9A—C9	173.8 (7)
C4'—C3'—C11'—F1'	−55 (4)	O1—C1—C9A—C4A	175.2 (4)
C2'—C3'—C11'—F2'	−61 (4)	C2'—C1—C9A—C4A	6(3)
C4'—C3'—C11'—F2'	60 (4)	C2—C1—C9A—C4A	−6.8 (9)
C2'—C3'—C4'—C4A	−64 (5)	C4A—N1—C10A—C8A	−1.5 (7)
C11'—C3'—C4'—C4A	174 (3)	C4A—N1—C10A—C5	179.4 (4)
C10A—N1—C4A—C9A	0.7 (7)	C8—C8A—C10A—N1	−179.6 (4)
C10A—N1—C4A—C4	179.2 (7)	C9—C8A—C10A—N1	1.2 (7)
C10A—N1—C4A—C4'	−168 (3)	C8—C8A—C10A—C5	−0.4 (7)
C3—C4—C4A—N1	154.8 (6)	C9—C8A—C10A—C5	−179.6 (4)
C3—C4—C4A—C9A	−26.7 (11)	C6—C5—C10A—N1	−179.4 (4)
C3—C4—C4A—C4'	77 (10)	Br1—C5—C10A—N1	−1.5 (6)
C3'—C4'—C4A—N1	−152 (2)	C6—C5—C10A—C8A	1.4 (7)
C3'—C4'—C4A—C9A	40 (5)	Br1—C5—C10A—C8A	179.3 (3)
C3'—C4'—C4A—C4	−43 (7)		
